# Concerning Clothes

**Published:** 1920-10-02

**Authors:** 


### CONCERNING CLOTHES.

There is no more fascinating chapter in Professor Leonard Hill's recent publication upon the science of ventilation and open-air treatment than that appearing under the above brief but explicit title. It matters not whether the reader's first interest is to find scientific and indisputable experimental evidence in support of the more recent teachings in hygiene, or whether he seeks simply some common generalised advice upon a homely but nevertheless vital subject.

In either case he will find his attention firmly held, for the author possesses that rare gift of making a highly technical essay readable as well as accurate, and of bringing its teaching well within the understanding of the least specialised health worker in the country. We note that the popular press has already made somewhat scanty reference to these chapters, but never was there a publication of the Medical Research Council which deserved greater public prominence and more direct representation of its moral to the public at large.

The hard-and-fast rules of hygiene laid down in the class-room, the welfare centre, and the hospitals themselves—the text-book "hygiene" of the past few years—have served their purpose well, and will in a limited sense continue to do so as each succeeding generation has them more thoroughly pounded into it. But what pity it is that more colour, the broader outlook, and the wealth of world-wide illustrations cannot be brought into our future citizens' instruction. Read but the first few pages of Professor Hill's work and by comparison the flatness of our accepted teaching is strikingly apparent.

To quote his own quotation: "Clothes gave us individuality, distinctions, social polity; clothes have made men of us, they are threatening to make clothes-screens of us"—a true summary indeed of the situation in highly civilised Europe. Yet Darwin has long ago described the endurance by the naked inhabitants of Tierra del Fuego of a winter climate more severe than our own—the woman with the babe at her breast, both naked, and the sleet falling and melting on them. Stunted in growth were these people, gaining food only by unremitting toil, committing, indeed, infanticide to keep the population down to the food supply. Yet Nature, by making habit omnipotent and its effects hereditary, has fitted the Fuegians to the climate and productions of their miserable country!

Let us remember the all too common type of town dweller, who complains that he always feels cold, whose nursery training has instilled into him the fear of cold, draught, and wet feet, and who, loaded with clothes, daily encourages a once perfect heat-regulating mechanism to atrophy from disuse. Compare him with the average ship's officer, who faces a night watch on the winter seas, wearing clothes not one jot more heat-retaining than his own. The channel swimmer—coated with grease, it is true—has stood exposure to sea-water for some twenty-three hours. And the cooling power of water is fourteen times greater than air! What range of adaptability of the heat mechanism and natural defence against cold must exist between the townsman, the swimmer, and the savage!

We like and agree with the observation of the Scot practitioner—a man of mature experience—who wrote: "The longer I work among people, the more I find cases of individuals who habitually go with little clothing and still show the greatest immunity to those diseases commonly attributed to the influence of cold." And yet the children in the elementary schools are reported as wearing as many as thirteen different articles of clothing.

Would that space allowed of a wider sketch of the "disability produced by clothes"! But enough has been said, perhaps, to illustrate our meaning. There are two sides to every question, and clothing requirements, as every other domestic consideration, are controlled by a thousand minor circumstances. The short-haired camel of Egypt will grow a long hairy coat in Thibet; but Nature produces such changes up to the limit of efficiency, and no shade more. Can we not imitate her rulings a little more closely than is habitually done to-day?

