# Addendum

**Published:** 1921-03-26

**Authors:** 


					Addendum.
AUUCIiUUin.
J? l'lr a|'s\v(>i- last week to a correspondent ? gho?^
h?v? i ' hca<1 <)f " Irish Nurses' Association, ?, CRb
//? I. x>on stated that the address of the lUar<?,
Dullli.f ? Nursi,1ff' Ltd-) >s 54 Fitzwillin.nl Sq?a

				

